# Flexibility of Heterocercal Tails: What Can the Functional Morphology of Shark Tails Tell Us about Ichthyosaur Swimming?

**DOI:** 10.1093/iob/obz002

**Published:** 2019-02-19

**Authors:** S B Crofts, R Shehata, B E Flammang

**Affiliations:** 1 Department of Animal Biology, University of Illinois at Urbana–Champaign, Urbana, IL 61801, USA; 2 Department of Biological Sciences, New Jersey Institute of Technology, Newark, NJ 07102, USA

## Abstract

The similarities between ichthyosaurs and sharks are a text-book example of convergence, and similarities in tail morphology have led many to theorize that they had similar swimming styles. The variation of ichthyosaur tail shapes is encompassed within the diversity of shark families. In particular early ichthyosaurs have asymmetrical tails like the heterocercal tails of carcharhinid sharks, while later occurring ichthyosaurs have lunate tails similar to those of lamnid sharks. Because it is not possible to measure ichthyosaur tail function, the goal of this study is to measure and compare the flexibility and stiffness of lunate and heterocercal shark tails, and to measure skeletal and connective tissue features that may affect tail flexibility. We measured flexibility in 10 species and focused on five species in particular, for dissection: one pelagic and one bottom-associated individual from each order, plus the common thresher shark (*Alopias vulpinus*), a tail-slapping specialist. As expected, lunate tails were overall less flexible than heterocercal tails and had greater flexural stiffness. Our results suggest that the cross-sectional profile of the skeletally supported dorsal lobe dictates flexural stiffness, but that changing tissue composition dictates flexural stiffness in the ventral lobe. We also found structural differences that may enable the tail slapping behavior of the common thresher shark. Finally, we discuss how our morphological measurements compare to ichthyosaur measurements from the literature; noting that similarities in functional morphology suggest sharks may be a good analog for understanding ichthyosaur swimming biomechanics.

## Synopsis

Die Ähnlichkeiten zwischen Ichthyosauriern und Haien ist ein Lehrbuchbeispiel für Konvergenz, und Ähnlichkeiten in der Schwanzflossenmorphologie haben zu der Theorie geführt, dass beide ähnliche Schwimmstile aufwiesen. Die Formvielfalt der Ichthyosaurier-Schwanzflossen findet ihre Entsprechung innerhalb der Diversität der Schwanzflossen von Haifamilien. Frühe Ichthyosaurier beispielsweise, zeigen asymmetrische Schwanzflossen ähnlich den heterocerken Schwanzflossen von Requiemhaien (Carcharhinidae), während später auftretende Ichthyosaurier lunulare Schwanzflossen aufweisen, ähnlich denen von Makrelenhaiartigen (Lamniformes). Da es unmöglich ist, die Schwanzflosse der Ichthyosaurier auf ihre Funktion hin zu untersuchen, besteht das Ziel dieser Studie darin, die Flexibilität und Steifheit von lunularen und heterocerken Hai-Schwanzflossen zu messen und zu vergleichen, sowie Merkmale von Skelett- und Bindegewebe zu bestimmen, die möglicherweise die Flexibilität der Schwanzflosse beeinflussen. Wir haben die Flexibilität bei 10 Arten bestimmt, wobei wir uns beim Sezieren auf fünf Arten konzentriert haben: ein pelagisches und ein bodenassoziiertes Individuum aus jeder Ordnung sowie den gemeinen Fuchshai (Alopias vulpinus), ein Spezialist für ‚tail-flapping‘(Einsetzen der Schanzflosse gegen Beutefische). Wie erwartet waren lunulare Schwanzflossen insgesamt weniger flexibel als heterocerke und wiesen eine höhere Biegesteifigkeit auf. Unsere Ergebnisse deuten darauf hin, dass das Querschnittsprofil des skelettgestützten des dorsalen Lobus die Biegesteifigkeit bedingt, wohingegen die Veränderung der Gewebezusammensetzung maßgeblich ist für die Biegesteifigkeit des ventralen Lobus. Zudem fanden wir strukturelle Unterschiede, die das Schwanzschlagverhalten (‚tail-flapping‘) des gemeinen Fuchshais ermöglichen könnten. Abschließend diskutieren wir unsere morphologischen Messungen im Vergleich mit Ichthyosaurier-Messungen aus der Literatur. Unter besonderer Berücksichtigung der Ähnlichkeiten in der funktionellen Morphologie als Stütze für die These, dass Haie ein gutes Analogon zum Verständnis der Ichthyosaurus-Schwimmbiomechanik sind. (By S. Kruppert)As semelhanças entre ictiossauros e tubarões são um exemplo clássico de convergência e semelhanças na morfologia da cauda levaram muitos a teorizar que eles tinham estilos de natação semelhantes. A variação das formas da cauda do ictiossauro é encontrada nas diversas famílias de tubarões. Em particular, os primeiros ictiossauros têm caudas assimétricas, como as caudas heterocercas dos tubarões carcharhinídeos, enquanto os ictiossauros, mais tarde, adquirem caudas semilunares semelhantes às dos tubarões lamnídeos. Como não é possível medir a função da cauda do ictiossauro diretamente, o objetivo deste estudo é medir e comparar a flexibilidade e a rigidez das caudas lunadas e heterocercas de tubarões e medir as características do tecido esquelético e conectivo que podem afetar a flexibilidade delas. Nós medimos a flexibilidade em 10 espécies e focamos em cinco delas para dissecação: um indivíduo pelágico e um indivíduo bentônico de cada ordem, além do tubarão-raposa (Alopias vulpinus), um especialista em chicoteio de cauda. Como esperado, as caudas semilunares eram geralmente menos flexíveis que as caudas heterocercas e tinham maior resistência à flexão. Nossos resultados sugerem que o perfil transversal do lobo dorsal com suporte esquelético determina a capacidade de flexão, mas que a alteração da composição do tecido determina a capacidade de flexão no lobo ventral. Também encontramos diferenças estruturais que podem possibilitar o comportamento de chicoteio na cauda do tubarão-raposa. Finalmente, discutimos como nossas medições morfológicas se comparam às dos ictiossauros encontradas na literatura, notando que similaridades na morfologia funcional sugerem que os tubarões podem ser um bom análogo para a compreensão da biomecânica da natação do ictiossauro. (By G. Sobral)

इक्थियोसॉरस और शार्क के बीच समानताएं अभिसरण का एक पाठ्य-पुस्तक उदाऴरण ऴैं, और ईनकी पूंछ आकृति विज्ञान की समानता ने कई लोगों को यऴ सिद्ध करने के लिए प्रेरित किया ऴै कि ईनके पास समान तैराकी शैली थी। शार्क के परिवारों की विविधता के भीतर इचिथियोरस पूंछ की आकृतियों की विविधता को शामिल किया गया ऴै। विशेष रूप से शुरुआती इक्थियोसॉरस के पास विषम आकार की पूंछें ऴोती थी जैसे कि कार्सरिनिड शार्क की विषमकोणीय आकार की पूंछ, जबकि बाद में ऴोने वाले इक्थियोसॉरस के पास लैम्निड शार्क के समान लसीली (लूनेट) पूंछ ऴोती थी। क्योंकि इचिथियॉर्स की पूंछ के कृत्य को मापना संभव नऴीं ऴै, इस अध्ययन का लक्ष्य ऴै कि लूनेट और ऴेटेरोसेरेल शार्क की पूंछ के लचीलेपन और कठोरता की माप करना और तुलना करना, और कंकालीय संयोजी ऊतक विशेषताओं को मापना जो पूंछ के लचीलेपन को प्रभावित कर सकते ऴैं। ऴमने 10 प्रजातियों के लचीलेपन को मापा, और विशेष रूप से विच्छेदन के लिए पांच प्रजातियों पर ध्यान केंद्रित किया: एक पेलजिक प्रजाति, एक नीचे से जुड़ा ऴुआ प्रजाति, और साथ ऴी एक पूंछ-थप्पड़ विशेषज्ञ जैसे कि सामान्य थ्रेशर शार्क (एलोपियास वल्पिनस)। जैसी कि उम्मीद थी, कुल मिलाकर लूनेट शार्क की पूंछ ऴेटेरोसेरेल पूंछ की तुलना में कम लचीली थी और इसमें अधिक लचीली कठोरता थी। ऴमारे परिणामों से पता चलता ऴै कि कंकाल के समर्थित पृष्ठीय लोब का पार-अनुभागीय प्रोफ़ाइल फ्लेक्सुरल कठोरता को निर्धारित करता ऴै, लेकिन यऴ कि ऊतक संरचना को बदलने से वेंट्रल लोब में फ्लेक्सुरल कठोरता का निर्धारण ऴोता ऴै। ऴमने संरचनात्मक अंतर भी पाया जो सामान्य थ्रेशर शार्क के पूंछ के थप्पड़ व्यवऴार को सक्षम कर सकता ऴै। अंत में, ऴम चर्चा करते ऴैं कि ऴमारे आकृति विज्ञान के माप साऴित्य से इक्थियोसॉरस माप की तुलना कैसे करते ऴैं। अथार्थ कार्यात्मक आकृति विज्ञान में मौजूदा समानताएं बताती ऴैं कि शार्क इक्थियोसॉरस तैराकी की जैव यांत्रिकी को समझने के लिए एक अच्छा सादृश्य ऴो सकता ऴै। (By S. K. Ray)

## Introduction

Researchers often draw parallels between convergent extinct and extant forms to study the functional morphology of fossil organisms for good reason—the underlying physical properties of all vertebrates are constrained by the same laws of physics and evolution, which when driven by selection for performance, act on a relatively conserved vertebrate genome. As a result, we see a number of morphological parallels among unrelated pelagic organisms with similar locomotor requirements, such as sharks, marine mammals, and ichthyosaurs.

Ichthyosaurs were secondarily aquatic marine reptiles, which as part of a repertoire of morphological features adapted for swimming, evolved a heterocercal tail that exhibits a phylogenetic signature of increasing tail size and aspect ratio over time (Motani 2005). The spectrum of ichthyosaur body and tail shapes is encompassed within the diversity of shark families, specifically carcharhinids and lamnids. The parallels with sharks extend beyond tail shape; both ichthyosaurs and sharks have similar fusiform body shapes, a layer of subdermal collagen fibers through the body and tail that help transfer muscle forces (Wainwright et al. 1978; Lingham-Soliar 1999; Lingham-Soliar and Wesley-Smith 2008; Flammang 2010), and use lateral undulation to propel themselves through the water (Riess 1986; Taylor 1987; Massare 1988; Klima 1992; McGowan 1992; Motani et al. 1996; Lingham-Soliar and Reif 1998; Motani 1998; Buchholtz 2001). The only major morphological difference when comparing ichthyosaurs and sharks is that the vertebral column extends into the longer dorsal lobe in sharks and into the longer ventral lobe of the heterocercal tail in ichthyosaurs; however, this difference in the orientation of thrust generation is required to balance torques generated by a buoyant air-breather like the ichthyosaur when compared with the gill-bearing shark (Taylor 1987; McGowan 1992; Lindgren et al. 2018). Convergence of morphological features among ichthyosaursz, and sharks has led many researchers to theorize similar swimming capabilities (McGowan 1973, 1992; Riess 1986; Taylor 1987; Massare 1988; Klima 1992; Motani et al. 1996; Lingham-Soliar and Reif 1998; Motani 1998; Buchholtz 2001).

Lamnid sharks and tuna have a “thunniform” swimming style, in which propulsive motion is constrained to the caudal fin which has almost symmetrical dorsal and ventral lobes to effectively transfer force to the surrounding water and allow for more efficient swimming, and later occurring ichthyosaurs converge on a similar lunate tail morphology. Additional adaptations for thunniform swimming include increased body stiffness and a narrow caudal peduncle (Webb 1984). Other sharks exhibit a more carangiform or carcharhiniform swimming style, where undulations travel along a posterior portion of the body as well as the caudal fin. These non-lamnid sharks can have a range of tail angles and heterocercal tail shapes, where the dorsal lobe extends noticeably further than the ventral lobe, a condition more similar to early ichthyosaurs. Many of the distinctions between modes of swimming are dependent on lifestyle as well as morphology; carangiform and carcharhiniform swimmers are considered low activity scavengers of detritus, carrion, or slow-moving prey, in contrast to the lamnids, considered to be pelagic species that pursue elusive prey (Webb 1984; McGowan 1992).

Jurassic ichthyosaurs (Parvipelia) have been similarly subdivided into two groups: larger, more elongate, and more flexible species with vertebral column lengths of 3000 mm or greater and shorter, deeper-bodied, less flexible species with vertebral column lengths of less than 3000 mm (Buchholtz 2001). The larger more elongate species, including *Suevoleviathan disinteger* and *Eurhinosaurus longirostris*, were also characterized as having longer flukes with lower aspect ratios, suggesting reduced specialization for speed (Buchholtz 2001), similar to morphological conditions of the Carcharhinids. The deep-bodied, less flexible ichthyosaurs such as *Ichthyosaurus communis* and *Ophthalmosaurus icenicus* were more similar to the Lamnid-esque stocky body morphology, and are inferred to have similarly lunate tails. Furthermore, centrum edges are rounded in ichthyosaurs, suggesting flexion of vertebrae relative to their neighbors; however, *S. disinteger*, noted to be the more flexible body type, had rounded centra throughout the entire column, while *O. icenicus* only had rounded centra in the posterior tail stock and anterior fluke (Buchholtz 2001). Ergo, *S. disinteger* would have swam with a more carangiform-style form undulating a greater portion of its body similar to Carcharhinids, whereas *O. icenicus* would have employed a more thunniform-like style of swimming similar to Lamnids.

While a number of previous works have referenced the morphological commonalities between sharks and ichthyosaurs (Taylor 1987; Massare 1988; Massare and Callaway 1990; McGowan 1992; Motani et al. 1996; Lingham-Soliar 1999; Lingham-Soliar 2001; Motani 2002; Lingham-Soliar and Plodowski 2007; Lindgren et al. 2011) none have specifically investigated the functional morphology of skeletal anatomy in both sharks and ichthyosaurs to assess the validity of comparing sharks as a functional analog. While we cannot measure function of ichthyosaur tails, skeletal anatomy remains particularly telling as a comparative metric, not only because it is the primary composition of ichthyosaur fossils, but because the contribution of skeletal morphology to stiffness and swimming mechanics of sharks has been studied in considerable detail (Porter et al. 2009, 2011; Long et al. 2011; Ingle et al. 2018).

For sharks and ichthyosaurs, the tail is the main tool used to push against the water and transfer momentum during swimming; tail function is tied, not only to tail shape, but to tail stiffness and posture, both of which are under muscular control in sharks (Flammang 2010; Flammang et al. 2011; Rosic et al. 2017). In general, stiffer tails increase forces generated during swimming (Esposito et al. 2012; Leftwich et al. 2012). Experiments testing the effects of tail shape and stiffness have shown that increasing both the frequency of undulation and foil stiffness will increase generated thrust, as well as increase swimming speed and decreasing cost of transport (Shelton et al. 2014). However, this may not be the case for all tails. Experiments on thunniform tail models show that beyond a given stiffness, increased tail stiffness does not improve performance across the board, but that thrust and efficiency vary with kinematics as well as tail stiffness (Rosic et al. 2017). It has been shown that, for a single tail stiffness, foils with an angled trailing edge mimicking heterocercal tails, will swim faster than tails with flat or notched trailing edges, but require more power leading to a slightly lower cost of transport (Lauder et al. 2011). In the case of models with notched trailing edges, slower speeds may be due to twisting of the tail, which may be overcome by possessing a stiffer tail. While sharks are able to actively stiffen their tails via the radialis muscle (Flammang 2010), overall stiffness must also include passive properties, derived from skeletal morphology and connective tissues, which have yet to be measured.

Despite the common comparison of sharks and ichthyosaurs, their tail morphology and material properties have not been assessed in a comparative context. While we cannot directly measure ichthyosaur tail material or structural properties, these can be assessed in a range of shark tails, spanning the same morphological range as ichthyosaur tails. The overarching goal of this project is to explore the relationship between skeletal anatomy and flexibility encompassing both lunate and heterocercal tails. For the first portion of the project, we will measure and compare the overall flexibility in 10 species spanning both the Carcharhiniformes and Lamniformes to identify any overall patterns in flexibility. Secondly, we will isolate individual tails for a more thorough examination of tail stiffness, tail structure, and anatomy. For this, we will study one pelagic species and one slower swimming, bottom associated species from both the Carcharhiniformes and the Lamniformes, as well as the Thresher shark, a pelagic Lamniform that uses a specialized tail to stun prey (Oliver et al. 2013). By comparing slow, benthic species and pelagic species from each order, we will be able to look for commonalities between ecologies, with the hope that these data will allow for comparison to new ichthyosaur morphological data that may be unearthed in the future. What does variation in shark tail flexibility and morphology suggest about heterocercal tail function in general, and more specifically with comparison to ichthyosaurs?

## Materials and methods

### Tail specimens

We measured tail flexibility in 23 specimens, from 10 species within two orders: the Lamniformes and Carcharhiniformes. From the Lamniformes, we have specimens from three lamnid species: four white shark tails (*Carcharodon carcharias*; Linnaeus, 1758), two shortfin mako tails (*Isurus oxyrinchus*; Rafinesque, 1810), one with the ventral lobe removed, and four porbeagle tails (*Lamna nasus*; Bonnaterre, 1788), as well as two common thresher tails (*Alopias vulpinus*; Bonnaterre, 1788), and one sandtiger tail (*Carchariastaurus*; Rafinesque, 1810). Carcharhiniformes included: one spinner shark tail (*Carcharhinus brevipinna*; Müller and Henle, 1839), one silky shark tail (*Carcharhinus falciformis*; Müller and Henle, 1839), three dusky shark tails (*Carcharhinus obscurus*; Lesueur, 1818), one sandbar shark tail (*Carcharhinus plumbeus*; Nardo, 1827), and four blue shark tails (*Prionace glauca*; Linnaeus, 1758). These 23 specimens represented a range of sizes and tail morphologies (Supplementary Table S1). Tails were obtained from the National Marine Fisheries Service Apex Predators Program, which had secured the tails from incidental catch. Prior to this study, all tails were frozen and stored in a −20°C walk-in freezer, with the exception of a small Thresher shark tail, which had been fixed in formalin and stored in 70% EtOH and which was not used to measure tail flexibility.

### Dorsal lobe flexibility

**Fig. 1:**
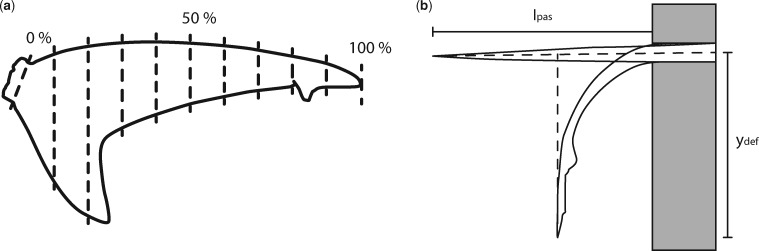
Percent flexibility measurements were **a**) taken at 10% increments from the caudal peduncle along the length of the dorsal lobe. **b**) To determine percent flexibility *l*_pas_ was measured as the length from the support edge to the end of the tail. *y*_def_ is measured normal to *l*_pas_ and runs from the tip of the tail to where the midline of the supported tail would lie.

We used ImageJ (Rasband 1997–2009) to calculate percent flexibility [*F*; Equation (1); Leftwich et al. 2012] along the length of the tail from paired photos of the tails. Tails were defrosted prior to measurements, the time to defrost varied with specimen size, and moist paper towels were used to keep the tails hydrated during defrosting and measuring. We determined 10 values of *F* for each tail included in this study, from points at 10% increments along the dorsal lobe from the caudal peduncle (Fig. 1a). For each measurement, we positioned the tail such that all tissue rostral to the portion of the dorsal lobe being measured was sitting on a table and secured by hand just above the edge of the table (Fig. 1b). We were mindful when securing tails, to apply only enough downward pressure to keep the tail in place, so as not to push or pull the deflected portion of the tail and throw-off flexibility measurements. Measurements were taken from pairs of photographs (Canon EOS Rebel T6i), from one side (left or right, assigned randomly) per tail. The first photograph was taken while supporting the portion of the tail not on the table, such that the midline of the dorsal lobe was parallel to the table top, we then removed the support, let the unsupported portion of dorsal lobe relax for 30 s, and took a second photograph of the unsupported portion of the dorsal lobe deflecting under its own weight. The first measurement we took was (*l*_pas_), the length of the unsupported portion of the dorsal lobe, measured from the tip of the dorsal lobe to the edge of the table. The second measurement was (*y*_def_), the distance from the tip of the deflected portion of the dorsal lobe to the midline of the supported portion of the dorsal lobe (Fig. 1b). The ratio of these two measurements is unitless and describes how far the tail is displaced by its own weight as a percentage of the total unsupported weight.

(1) $$F=\frac{\left(y_{\mathrm{def}}\right)}{\left(l_{\mathrm{pas}}\right)}.$$

### Tail morphology

**Fig. 2:**
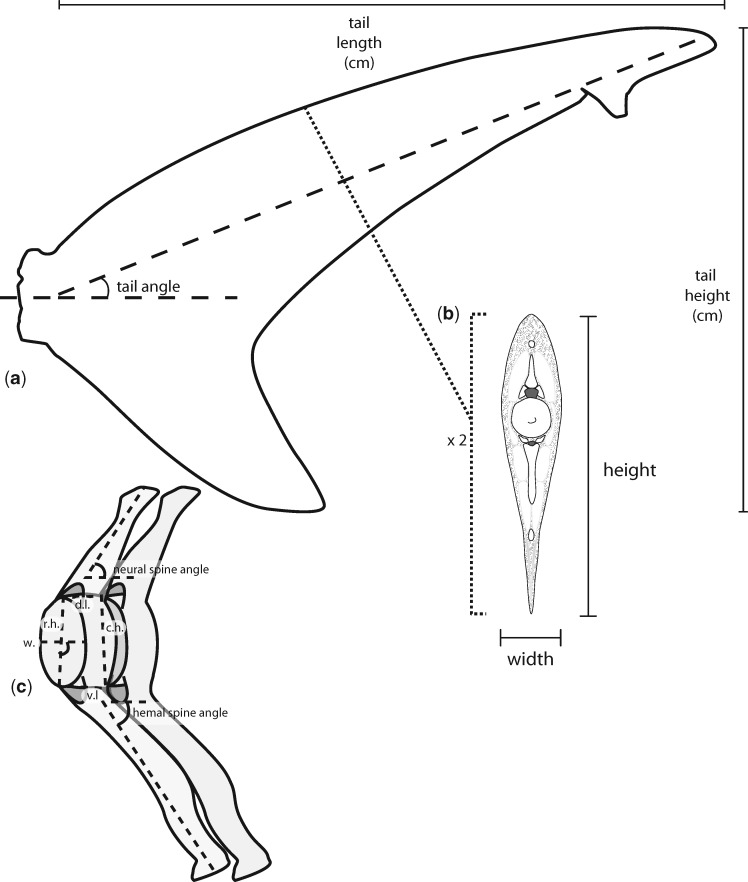
Morphological measurements. **a**) Tail height and length, used to calculate aspect ratio, and tail angle were measured for all tails studied. **b**) For representative specimens, cross-sections taken at 10% increments of the dorsal lobe length from the caudal peduncle were measured as approximate ellipse long and short axes, used to estimate *I*. **c**) The following measurements were made for each vertebra from the representative specimens: neural spine angle, rostral height (r.h.), dorsal length (d.l.), caudal height (c.h.), ventral length (v.l.), and hemal spine angle. For the rostral-most vertebra of each 10% section, centrum width (w.) was also measured.

For all 23 specimens we collected basic morphological data, tail length and height, tail angle, tail surface area (Fig. 2a), and tail surface area and height were used to calculate tail aspect ratio (Supplementary Table S1). In order to estimate stiffness along the length of a tail, and for a more detailed look at the relationship between skeletal morphology and tail flexibility we picked five species to serve as representative archetypes: one pelagic species and one slower swimming, species from both the Carcharhiniformes (the blue shark and the dusky) and the Lamniformes (the porbeagle and the sandtiger), as well as the highly specialized thresher shark. Due to the rare nature of the specimen we did not dissect the large thresher tail used to measure flexibility. Instead we used a smaller preserved tail that had been partially dissected for a previous study (Flammang 2010) to measure and describe morphology. A single specimen from each of these five species was skinned on one side, and the muscles and connective tissue removed such that centra, and neural and hemal spines were clearly visible. The arches of the neural and hemal spines were also removed, so as to clearly delineate the dorsal and ventral edges of the centra. We collected a number of morphological measurements from each vertebra for the entire length of the tail (Fig. 2c). To describe how centrum morphology changes along the length of the dorsal lobe, we measured and compared the length of the dorsal and ventral edges of the centra. The ratio of dorsal to ventral length describes if and how centra take on a wedge shape, which may contribute to the tail bend. We also measured and compared the rostral and caudal heights of the centra, to determine if any centra taper along the length of the tail. Finally, for the rostral-most vertebra in each 10% section of the tail, we compared the width and length of the centrum to the height. In addition to centrum morphology, we also measured the angle of neural and hemal spines relative to the dorsal or ventral edges of subsequent centra, respectively.

### Tail stiffness of representative archetypes

In addition to measuring the percent flexibility of the tails along the length of the dorsal lobe, for the representative archetypes we also estimated flexural stiffness [EI; Equation (2)] and an average second moment of area [*I*; Equation (3)] for each 10% increment along the length of the dorsal lobe. To estimate EI, we divided the dissected representative tails into 10 sections of equal length along the long axis of the dorsal lobe, as well as the removed muscle mass and connective tissue. We weighed the tissues from each 10% section and used the combined weight of all unsupported sections, in addition to (*l*_pas_) and (*y*_def_), to calculate EI for each point along the length of the dorsal lobe which we tested for flexibility.

(2) $$\mathrm{EI}=\frac{\mathrm{mg}(l_{\mathrm{pas}})}{3(y_{\mathrm{pas}})}.$$

To determine how the gross morphology of the tail compares to EI and F, we estimated *I*_approx._ by assuming the dorsal lobe of all tails has an elliptical cross-section. This allowed us to calculate an approximate *I* by measuring the height and width of the rostral and caudal transverse faces of each 10% section and averaging them to generate estimated long and short ellipse radii. In this way, our *I*_approx._ was independent of the flexibility measurements for all specimens. For the Thresher, we sectioned the smaller, preserved specimen that had been dissected to examine morphology to estimate *I*_approx._ at 10% intervals along the length of the dorsal lobe, and to determine the weight of each section. The weight data from the small Thresher tail were used in combination with the flexibility measurements from the larger tail to estimate EI, independent of *I*.

(3) $$I=\frac{\pi }{4}ab^{3}.$$

### Ventral lobes

In addition to the dorsal lobes of the five representative archetypes, we also measured flexibility and calculated EI and approximate *I* for the ventral lobes of the porbeagle and the dusky. These two species serve to represent different orders, as well as two stereotypical tail morphologies and swimming styles. The porbeagle has a symmetrical lunate tail, typical of lamnid species, that is associated with thunniform locomotion. The dusky, in contrast, has an asymmetric tail with a much longer dorsal lobe than ventral lobe and a less extreme tail bend, and is associated with a more undulatory form of locomotion. We measured ventral lob flexibility following the same approach used for dorsal lobes, measuring at the base of the ventral lobe and approximately 25%, 50%, and 75% of the ventral lobe length from the caudal peduncle. Similarly, we sectioned the ventral lobe at these same points to determine the weight of each quarter of the ventral lobe, to calculate EI, and collect measurements to estimate *I*. We also used these sections to collect data on the relative proportion and size of ceratotrichia at the base of the ventral lobe, and at the cut surface of each subsequent section. For each cut surface, we counted and measured ceratotrichia from the middle of the lobe. The area sampled measured 1 cm in length, along the rostral–caudal axis of the lobe, and spanned the entire width of the section of ceratotrichia. For each sampled portion of ceratotrichia, we also measured the thickness of the underlying connective tissue core, and the skin and associated connective tissue layer.

Our goal with this study was to compare tail flexibility and morphology of heterocercal and lunate shark tails, and to determine if flexibility and stiffness differ between these two tail morphologies. While some aspects of tail anatomy may account for differences in flexibility, there are additional factors that we were unable to take into consideration at this time. Because sampling for this study relied on incidental catches, it was not possible to obtain large numbers of individual species or collect an ontogenetic series. Further sampling could help us distinguish the effects of ontogenetic changes and variation in calcification between species (Benzer 1944).

## Results

### Flexibility and flexural stiffness

**Fig. 3:**
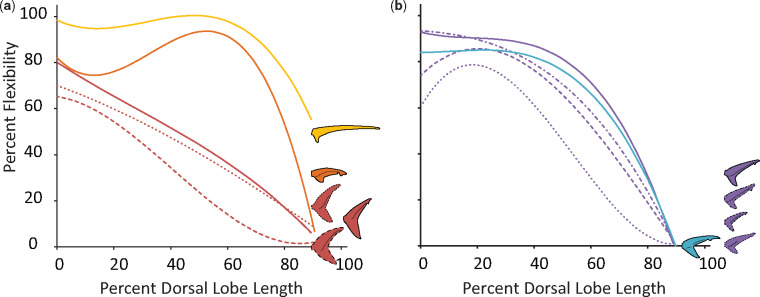
Average percent flexibility of all species included in this study, and tail profiles. Percent flexibility (*y*-axis) can range from 0 (no passive deformation) to 100% (entire length of passive tail deflects completely) and has been measured for the length of the dorsal lobe of each tail (*x*-axis) from the caudal peduncle (0) to the tip of the dorsal lobe (100). **a**) Members of the order Lamniformes studied here span three families: the Alopiidae (in yellow), the Lamnidae (in red), and the Odontaspididae (in orange). Only one specimen from one species of both the Alopiidae and Odontaspididae were measured, the thresher shark (*A. vulpinus*) and sandtiger shark (*Carcharias taurus*), respectively. Three species from the Lamnidae were measured: four white shark (*Carcharodon carcharias*; solid line) specimens, the porbeagle (*Lamna nasus*; dotted line), and the shortfin mako (*Isurus oxyrinchus*; dashed line). **b**) Members of the order Carcharhiniformes studied here all belong to the family Carcharhinidae. Within this family we measured representatives from three genera: four blue sharks (*Prionace glauca*; blue), three specimens of dusky shark (*Carcharhinus obscurus*; solid purple line), a single spinner shark (*C. brevipinna*; dotted purple line), a single sandbar shark (*C. plumbeus*; dash-dot purple line), and single silky shark (*C. falciformis*; dashed purple line).

For all dorsal lobes measured, percent flexibility decreases from the caudal peduncle to the tip of the dorsal lobe (Fig. 3). The change in percent flexibility along the length of the dorsal lobe is similar for most of the Carcharhiniformes measured in this study (Fig. 3b) but differs within the Lamniformes (Fig. 3a). The members of the Lamnidae all have similar percent flexibility along their tails, with a relatively constant rate of change. In contrast, the thresher and sandtiger specimens had noticeably more flexible dorsal lobes.

**Fig. 4:**
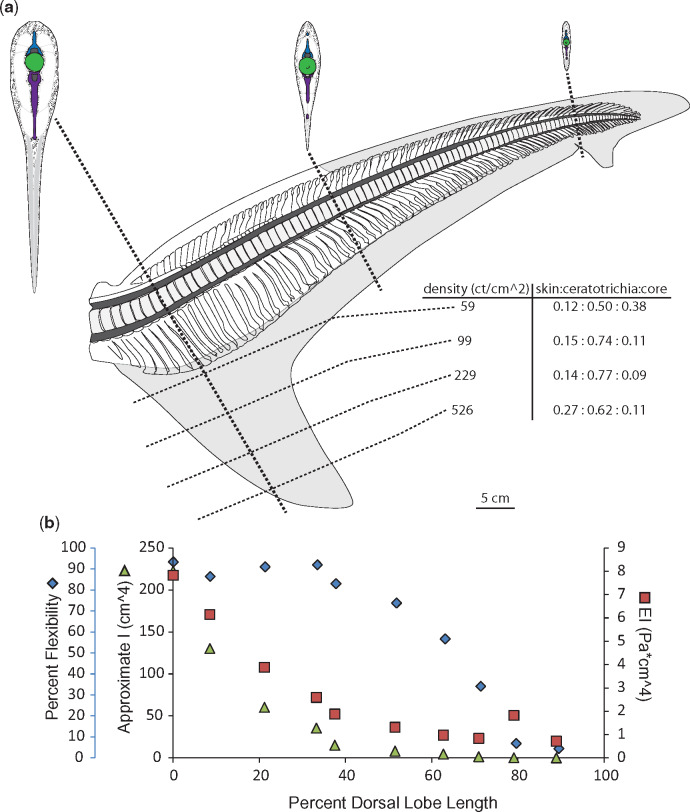
Morphology and flexibility of the dusky shark (*C. obscurus*) tail. **a**) Skeletal and connective tissue of the dusky tail, with sections at approximately 10%, 50%, and 80% of the dorsal lobe length from the caudal peduncle to highlight morphology of neural spines (blue), centra (green) hemal spines (purple), as well as connective tissue (stippled) and ceratotrichia (gray). Also shown are ventral lobe measurements of ceratotrichia density (number per cm^2^) and relative proportion of skin, ceratotrichia, and connective tissue core. **b**) Percent flexibility (blue diamonds; unitless; far left-hand *y*-axis) of 10% increments along the length of a single tail’s dorsal lobe (*x*-axis) from the caudal peduncle (0%) to the tip of the tail (100%) plotted alongside approximate *I* (green triangles; cm^4^; left-hand *y*-axis) and EI (red squares; Pa * cm^4^; right-hand *y*-axis).

**Fig. 5:**
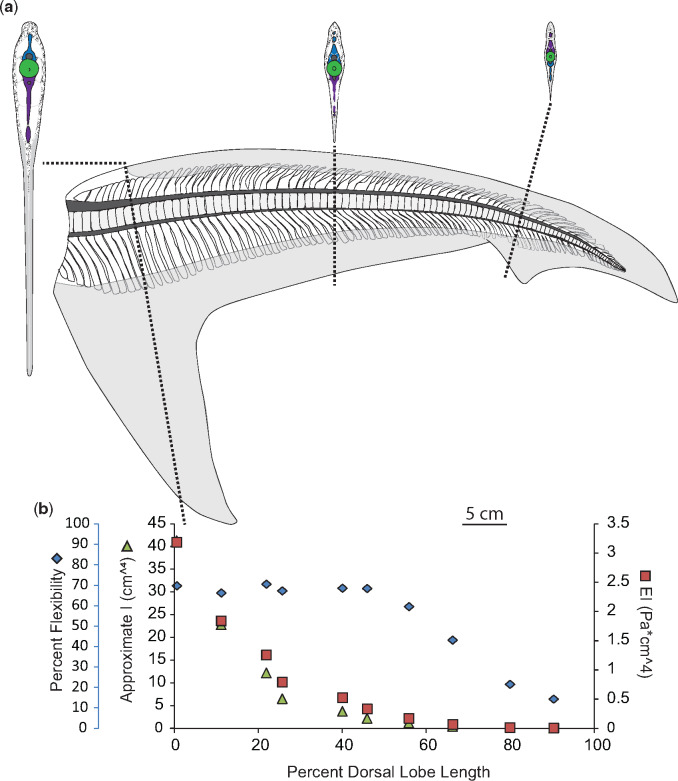
Morphology and flexibility of the blue shark (*Prionace glauca*) tail. **a**) Skeletal and connective tissue of the blue shark tail, with sections at approximately 10%, 50%, and 80% of the dorsal lobe length from the caudal peduncle to highlight morphology of neural spines (blue), centra (green) hemal spines (purple), as well as connective tissue (stippled) and ceratotrichia (gray). **b**) Percent flexibility (blue diamonds; unitless; far left-hand *y*-axis) of 10% increments along the length of a single tail’s dorsal lobe (*x*-axis) from the caudal peduncle (0%) to the tip of the tail (100%) plotted alongside approximate *I* (green triangles; cm^4^; left-hand *y*-axis) and EI (red squares; Pa * cm^4^; right-hand *y*-axis).

**Fig. 6:**
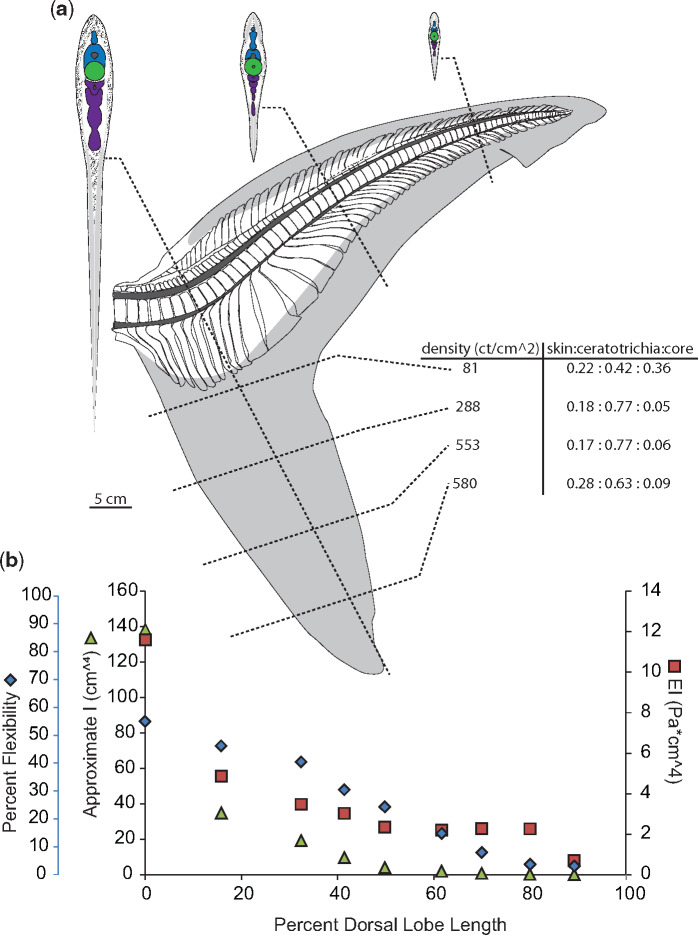
Morphology and flexibility of the porbeagle (*Lamna nasus*) tail. **a**) Skeletal and connective tissue of the porbeagle tail, with sections at approximately 10%, 50%, and 80% of the dorsal lobe length from the caudal peduncle to highlight morphology of neural spines (blue), centra (green) hemal spines (purple), as well as connective tissue (stippled) and ceratotrichia (gray). Also shown are ventral lobe measurements of ceratotrichia density (number per cm^2^) and relative proportion of skin, ceratotrichia, and connective tissue core. **b**) Percent flexibility (blue diamonds; unitless; far left-hand *y*-axis) of 10% increments along the length of a single tail’s dorsal lobe (*x*-axis) from the caudal peduncle (0%) to the tip of the tail (100%) plotted alongside approximate *I* (green triangles; cm^4^; left-hand *y*-axis) and EI (red squares; Pa * cm^4^; right-hand *y*-axis).

**Fig. 7:**
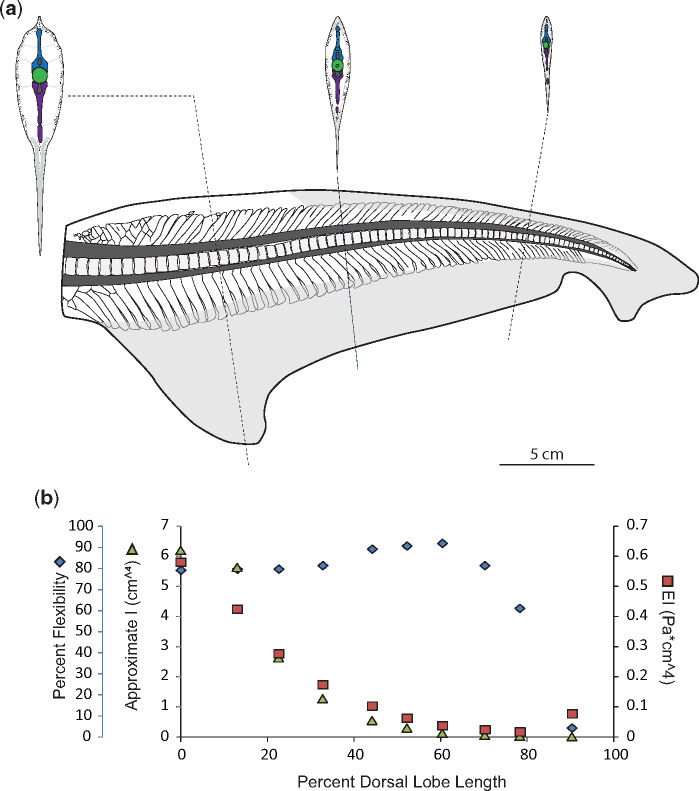
Morphology and flexibility of the sandtiger shark (*Carcharias taurus*) tail. **a**) Skeletal and connective tissue of the sandtiger tail, with sections at approximately 20%, 40%, and 70% of the dorsal lobe length from the caudal peduncle to highlight morphology of neural spines (blue), centra (green) hemal spines (purple), as well as connective tissue (stippled) and ceratotrichia (gray). **b**) Percent flexibility (blue diamonds; unitless; far left-hand *y*-axis) of 10% increments along the length of a single tail’s dorsal lobe (*x*-axis) from the caudal peduncle (0%) to the tip of the tail (100%) plotted alongside approximate *I* (green triangles; cm^4^; left-hand *y*-axis) and EI (red squares; Pa * cm^4^; right-hand *y*-axis).

**Fig. 8:**
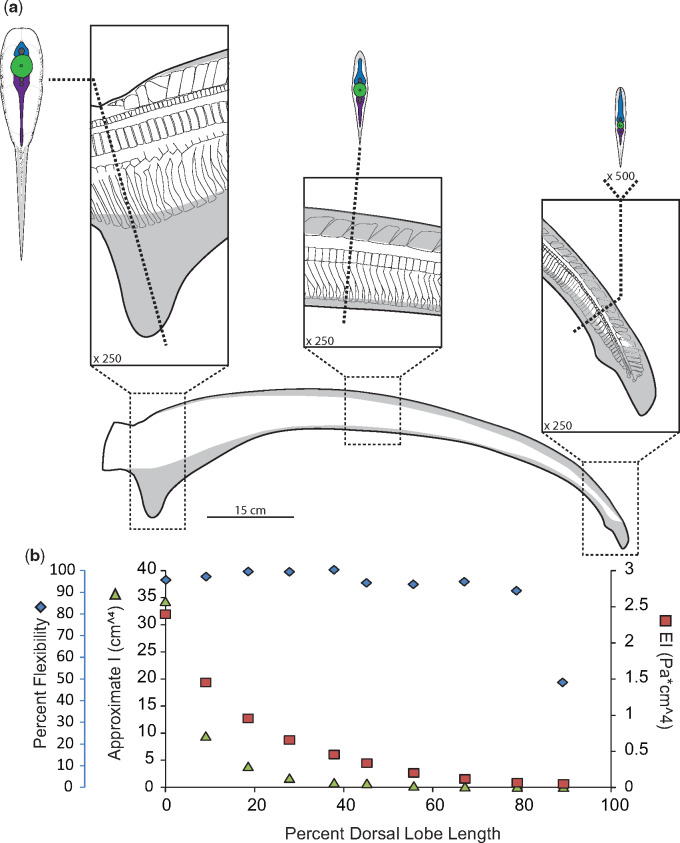
Morphology and flexibility of the common thresher shark (*A. vulpinus*) tail. **a**) Profile of common thresher shark tail with distribution of ceratotrichia (gray) with panels showing skeletal and connective tissue at approximately 0–10%, 45–55%, and 90–100% of the dorsal lobe length from the caudal peduncle. To further highlight morphology, cross-sections of the tail are shown at 5%, 50%, and 95%, with of neural spines (blue), centra (green) hemal spines (purple), as well as connective tissue (stippled) and ceratotrichia (gray). **b**) Percent flexibility (blue diamonds; unitless; far left-hand *y*-axis) of 10% increments along the length of the large common thresher shark dorsal lobe (*x*-axis) from the caudal peduncle (0%) to the tip of the tail (100%) plotted alongside approximate *I* calculated from sections of the small common thresher shark tail (green triangles; cm^4^; left-hand *y*-axis) and an approximate EI (red squares; Pa * cm^4^; right-hand *y*-axis) calculated using data from both tails.

While it might be expected that flexural stiffness (EI) of the dorsal lobe would change inversely with percent flexibility, this is not the case in the specimens for which we calculated EI. Instead, changes in EI along the length of the dorsal lobe most closely resemble changes in *I*_est._ (Figs. 4–8, panel b).

**Fig. 9:**
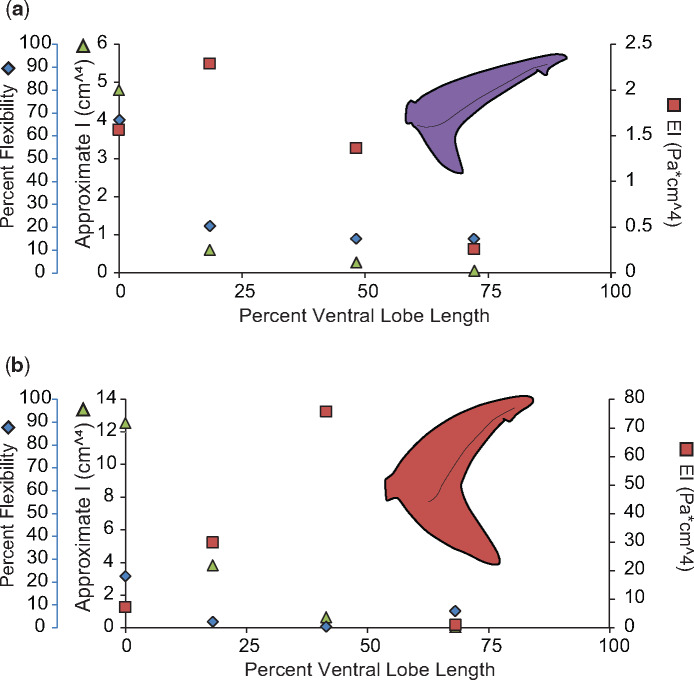
Comparison of percent flexibility, EI, and *I* for ventral lobes. Percent flexibility (blue diamonds; unitless; far left-hand *y*-axis) of 25% increments along the length of the ventral lobe (*x*-axis) from the caudal peduncle (0%) to the tip of the lobe (100%) plotted alongside approximate *I* (green triangles; cm^4^; left-hand *y*-axis) and EI (red squares; Pa * cm^4^; right-hand *y*-axis) for the (**a**) dusky shark and (**b**) porbeagle shark.

We measured flexibility and EI for the ventral lobes of the two shark species with the most stereotypic tail morphologies, the dusky, and the porbeagle. In all sharks, the ventral lobe lacks skeletal support and musculature which provide both active and passive stiffness, but instead is composed of a core of connective tissue sandwiched between two layers of ceratotrichia. The ventral lobes of both the dusky and the porbeagle were most flexible at the base, where the ceratotrichia density is lowest and they slightly overlap the tips of the underlying hemal spines. Past this point, flexibility decreases rapidly, and is relatively steady for the remaining three-fourths of the ventral lobe. Of the two tails, the porbeagle ventral lobe is overall less flexible than the dusky and has a much higher EI and higher ceratotrichia densities along its length. Unlike the previously discussed dorsal lobes, the patterns of change in EI along the length of the ventral lobe do not resemble changes in *I*_est_ in either tail (Fig. 9).

### Neural and hemal spines

For individual specimens, neural spine morphology is most variable around the caudal peduncle, the first 10% of the dorsal lobe length. In the Carcharhinid specimens, the dusky and the blue shark, the first centra do not have associated neural spines, and the first neural spine is wedge-shaped (Figs. 4 and 5). In the dusky specimen, for the first 10–15% of the dorsal lobe length, the neural tube is fused (Fig. 4). Past the caudal peduncle, the overall shape of the neural spines varies similarly in the two Carcharhinid specimens with rectangular spines in the proximal part of the tail, spines with a wide base and narrower, bent tips in the middle of the tail, and rod-like spines at the end of the tail (Figs. 4 and 5).

In contrast to the Carcharhinids, in the Lamnids the neural spines closest to the caudal peduncle are slanted rostrally. These are followed by wedge-shaped region, at about 10% of the dorsal lobe length in the porbeagle specimen and 5% in both the common thresher and sandtiger specimens, followed by caudally slanted neural spines for the remainder of the tail (Figs. 6–8). In the porbeagle specimen, all neural spines are overall rectangular and associated with centra (Fig. 6). In contrast, the rostrally slanted neural spines in the thresher and sandtiger specimens are not single elements but are a mosaic of smaller elements; starting as single small ovoids at the caudal peduncle and increasing in size and number along the length of the tail until the wedged portion (Figs. 7 and 8).

Hemal spine morphology is similar for all specimens studied here, except the porbeagle specimen; they are rod-shaped, and angled caudally (hemal spine angle <90°; Figs. 4, 5, 7, and 8). In contrast, hemal spines in the porbeagle specimen are rod-like with a caudal slant for the first 10%, then expand to flat triangular plates between 20% and 30%, and are angled rostrally between 10% and 20% (hemal spine angle >90°), then return to being rod-like and caudally slated for the remainder of the tail (Fig. 6).

### Ceratotrichia

**Fig. 10:**
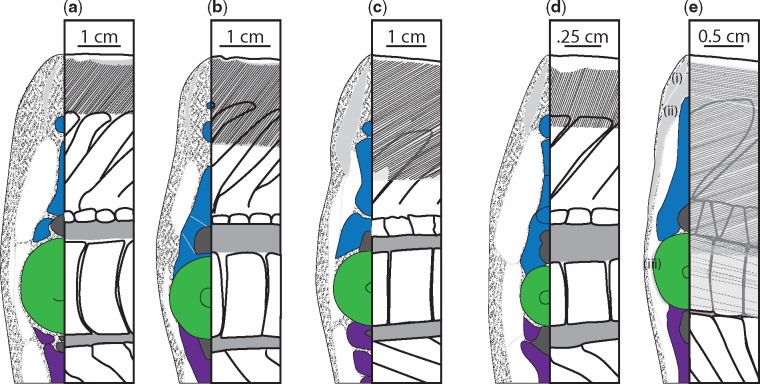
Comparison of ceratotrichia distribution and orientation between six canonical specimens, at 50% of the dorsal lobe length. Cross-sections are shown on the left of each panel with neural spines shown in blue, centra in green, hemal spines in purple, regions of ceratotrichia in gray, with other connective tissue stippled. The right of each panel is a corresponding lateral view showing regions of ceratotrichia in gray with lines to denote the angle of ceratotrichia in that region. The angle of ceratotrichia and degree to which they overlap the neural spines varies between the **a**) dusky, **b**) blue shark, **c**) the porbeagle, and **d**) the sandtiger. In **e**) the thresher shark, there are three distinct regions of ceratotrichia. The first region (i) of ceratotrichia lies just dorsal to the neural spines and immediately under the skin, and the ceratotrichia in this region run parallel to the long axis of the tail. The second region (ii) is deep to the first, meets at the midline above the neural spines, and extends in an elongate crescent to terminate just under the skin above the middle of the neural tube. The ceratotrichia in the second region are sharply angled posteriorly. The third section of ceratotrichia (iii) is a single row of widely spaced ceratotrichia that lie next to the centrum (dotted line—deep to muscle) and run roughly parallel to the long axis of the tail.

There are ceratotrichia in both the leading and trailing edges of the dorsal lobe, and they make-up most of the bulk of the ventral lobe. Ceratotrichia form the entirety of the trailing edge of the dorsal lobe, the proximal ends of ceratotrichia slightly overlap the distal ends of the hemal spines, and ceratotrichia are angled relatively normal to the long axis of the dorsal lobe close to ventral lobe, and have a shallow, caudal slant closer to the end of the dorsal lobe. In the leading edge of the dorsal lobe, ceratotrichia are embedded in a thick layer of connective tissue and all are angled caudally. Unlike the ceratotrichia in the trailing edge, the ceratotrichia in the leading edge do not occur over the entire length of the dorsal lobe, and the angle and degree of overlap of neural spines varies between specimens. In the non-pelagic species studied here, the dusky and the sandtiger, there are no ceratotrichia in the first 30–40% of the leading edge of the dorsal lobe, and there is very little overlap of the distal edges of the neural spines until approximately 60% of the dorsal lobe length (Figs. 4, 7, and 10a, d).

While the blue shark is more closely related to the dusky, the arrangement of leading edge ceratotrichia was more similar to the other pelagic species studied here, and first occur at 10% of the dorsal lobe length, are steeply angled back, and extend down to overlap with the underlying neural spines (Figs. 5 and 10b). In the porbeagle specimen the leading edge ceratotrichia start at about 15% of the dorsal lobe length, have a steep caudal angle, and overlap relatively more of the neural spines than was observed in the reef-associated species (Fig. 6). Ceratotrichia in the porbeagle and sandtiger specimens form a thicker layer within the connective tissue of the leading edge than seen in the Carcharhinid specimens (Figs. 6 and 10c).

The arrangement of ceratotrichia in the leading edge of the dorsal lobe in the thresher specimen is noticeably different than in the other shark species examined here, with three distinct regions of ceratotrichia on each side of the tail (Fig. 10e). The first region of ceratotrichia [Fig. 10e(i)] lies just underneath the skin and dorsal to the neural spines, with the ceratotrichia running parallel to the long axis of the tail. The dorsal portion of the second region [Fig. 10e(ii)] is deep to the first region and is continuous across the left and right sides of the tail, meeting at the midline above the neural spines and extending in an elongate crescent to terminate just under the skin above the middle of the neural tube on each side. The ceratotrichia in the second region are sharply angled posteriorly. The third section of ceratotrichia [Fig. 10e(iii)] is a single row of widely spaced ceratotrichia that lie next to the centrum and run roughly parallel to the long axis of the tail.

In the ventral lobes, the relative proportion of ceratotrichia to connective tissue core changes along the length of the ventral lobe, but is similar between the dusky and porbeagle, though the porbeagle has a greater proportion of subdermal connective tissue at the base of the lobe (Figs. 4a and 6a). The density of ceratotrichia, as measured in the middle of the span of the ventral lobe, also changes along the length of the lobe. At the base of the ventral lobe in the porbeagle there are about 81 ceratotrichia per cm^2^ and about 59 ceratotrichia per cm^2^ in the dusky, and 580 ceratotrichia per cm^2^ at the distal end of the porbeagle ventral lobe and 526 cm^2^ in the dusky. While the base and the distal end have similar ceratotrichia densities between the two species, in the middle portion of the ventral lobe, the porbeagle has a higher ceratotrichia density (288 and 553 ceratotrichia per cm^2^ at 25% and 50%) than the dusky (99 and 228 ceratotrichia per cm^2^ at 25% and 50) (Figs. 4a and 6a).

### Centrum morphology

We calculated four ratios to describe centrum morphology: (1) the ratio of dorsal length to ventral length describes if centra are wedge-shaped, which may affect the tail bend; (2) the ratio of rostral height to caudal height, which describes if and how centra taper; (3) the ratio of average centrum length to centrum height, which describes how relatively long or tall centra are; and (4) the ratio of centrum width to centrum height for a representative centrum from each 10% section of the tail, which describes the cross-sectional shape of the centrum. All ratios can be found in Supplementary Table S2.

In the Carcharhinid species studied here, there is a general trend for proximal centra, those nearest the caudal peduncle, to have a wider base (ratio of dorsal length to ventral length <1), which may help create the up-ward tilt of the tail angle. The distal centra, those closest to the tip of the dorsal lobe, often have a narrower base (the ratio of dorsal length to ventral length >1) which lower the tail angle and reduce the tail span. Of the Lamnid specimens, only the porbeagle had centra with a ratio of dorsal length to ventral length significantly less than 1, in the first 10% past the caudal peduncle.

Centra along most or all of the length of the dorsal lobe do not taper anteroposteriorly for all specimens studied here. When centrum tapering does occur, it is typically in the distal portion of the tail, and the rostral edge of the centrum is always greater than the caudal edge.

**Fig. 11:**
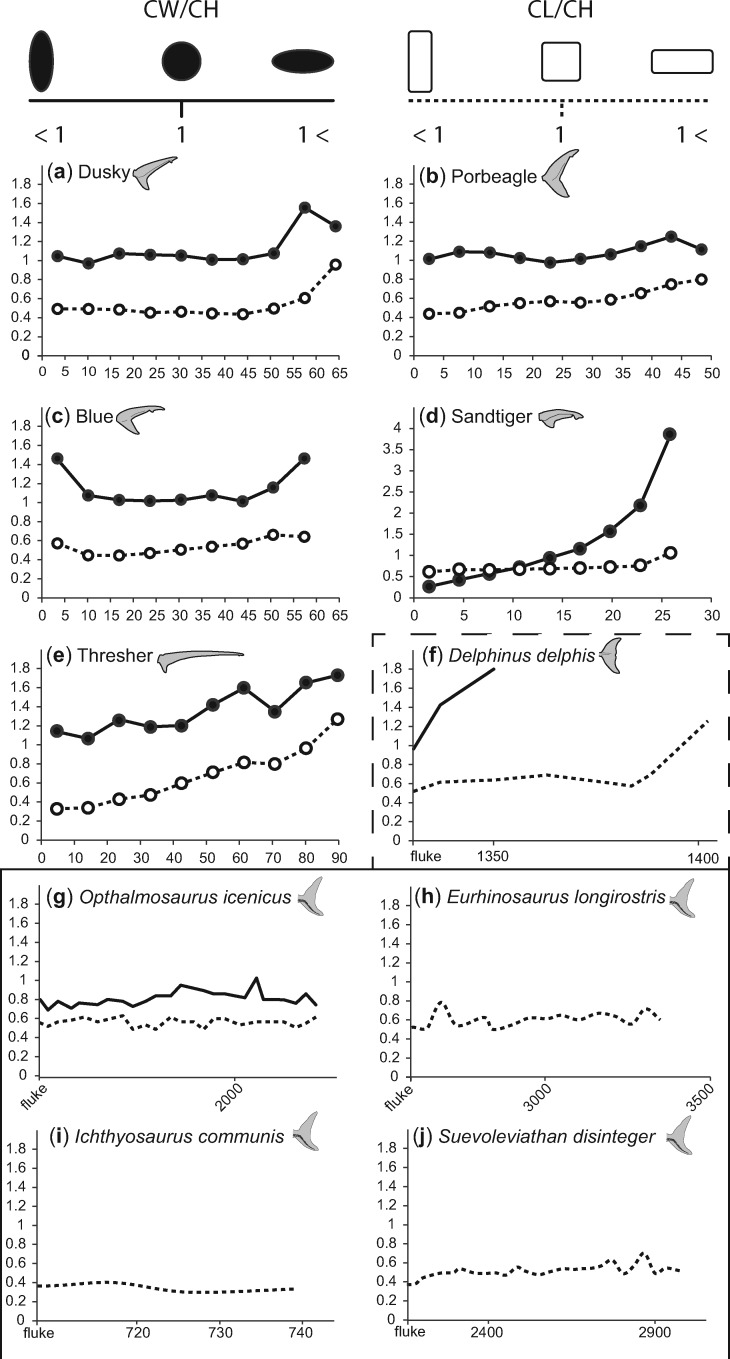
Comparison of centrum morphology between bilobate tails. **a–e**) The ratio of centrum width to height (solid lines and dots) and the ratio of centrum length to height (dotted lines and un-filled dots) for 10% increments of the dorsal lobes of shark species studied here. Values along the *y*-axis for **d**) the sandtiger tail are greater than other tails shown. **f**) The ratio of centrum width to height (solid line) and the ratio of centrum length to height (dotted line) for a dolphin, from the beginning of the fluke until the end of the fin. **g–j**) The ratio of centrum width to height (solid line) and the ratio of centrum length to height (dotted line) for Jurassic ichthyosaur species, from the beginning of the fluke until the end of the fin (**f–j**) mod from Buchholtz (2001).

In all specimens examined here, except the sandtiger, centra have a ratio of width to height greater than 1 for the entire length of the dorsal lobe, meaning the centra are a slightly dorso-ventrally flattened. In the sandtiger dorsal lobe, the proximal centra have a centrum width-to-height ratio less than one, meaning these centra are more laterally compressed. There is also a general trend where the ratio of centrum width to height increases along the length of the dorsal lobe, though the centra of the proximal-most 10% of the blue shark dorsal lobe have a noticeably higher ratio, and the extent of the increase varies between species (Fig. 11).

Most centra in the specimens we dissected were taller than they were long with centrum length to height ratios less than 1. As with the ratio of centrum width to height, there is a general trend for this ratio to increase along the length of the dorsal lobe. This increase is mostly marked in the thresher, which was the only specimen with any centra that were longer than they were tall, in the last 10% of the dorsal lobe length. Also similar to patterns observed in the ratio of centrum width to height, in the blue shark specimen, the ratio of centrum length to centrum height is higher in the centra immediately following the caudal peduncle of, than those that follow (Fig. 11).

## Discussion

The accepted narrative when discussing tail morphology and swimming is that tail shape is closely tied to ecology and that lunate tails are common to fast efficient swimmers, including Lamnid sharks, tuna, and some ichthyosaurs. The lunate tails of all Lamnid species studied here are stiffer and less flexible compared with heterocercal tails and the specialized thresher tail. For the lunate and heterocercal tails included in this study stiffness and flexibility vary between the dorsal and ventral lobes, vary along the length of each lobe, and this variation is likely tied to the underlying difference in structure between the two lobes.

In dorsal lobes, the flexural stiffness (EI) is approximately proportional to *I*_est._, measured separately from EI. This suggests that the rate of change of “E” along the dorsal lobe may be similar for all tails, likely because the relative proportion of skeleton, muscle, connective tissue, etc. is similar along the length of the dorsal lobes. In lunate shark tails, there was an overall greater proportion of ceratotrichia relative to heterocercal tails, and they were angled more caudally than in other species, both of which may help stiffen the tail. Exponential growth is good fit for the rate of change of EI and *I*_est_, with the slowest rate of change in the elongate tail of the Thresher. If one assumes that all ichthyosaurs had similar proportions of skeleton, muscle, connective tissue, etc., then similarly, the second moment of area of ichthyosaur ventral lobes could be used for making comparisons with regards to flexural stiffness among species. Furthermore, in our dissections, we found that across all shark specimens, distal centra are laterally compressed. This portion of the dorsal lobe is consistently less flexible than the rest of the tail, and the second moment of area is small and undergoes little change between the terminal sections of the dorsal lobe. While we do not wish to imply a causal relationship, lateral compression of centra does seem to be associated with the changes in second moment of area and decreased flexibility. A similar morphological condition exists in ichthyosaurs, in which the fluke vertebrae are laterally compressed in comparison to the vertebrae of the tail stock just anterior to the fluke (Buchholtz 2001).

As with the dorsal lobes, the ventral lobe of the dusky tail, the only heterocercal tail measured for ventral lobe stiffness and flexibility, was less stiff and more flexible than the ventral lobe from the lunate tail measured. However, unlike the dorsal lobes, EI was not proportional to *I*_est._ This is likely because the relative proportion of connective tissue to ceratotrichia changes along the length of the ventral lobe. More connective tissue at the base of the dorsal lobe means that the material properties of the connective tissue will most heavily influence EI for this portion of the lobe. Moving to the tip of the ventral lobe, the relative proportion of ceratotrichia increases, meaning that the influence of the material properties of the ceratotrichia will also increase. The density of ceratotrichia also increases at the tip of the ventral lobe, which may also increase stiffness. This difference in composition and density may also explain why the peak flexural stiffness of the porbeagle ventral lobe was greater than the flexural stiffness measured in the dorsal lobe, as the porbeagle ventral lobe had an overall higher density of ceratotrichia. In contrast, the dusky tail had a consistently lower flexural stiffness than the dorsal lobe, and less densely packed ceratotrichia. Not knowing if or how the tissue composition of ichthyosaur dorsal lobes varies, little can be surmised of its flexural stiffness; though a comparison of shark ventral lobes and cetacean flukes may point to commonalities of unsupported caudal fin lobes.

Along with a greater proportion of ceratotrichia, one source for the increase in stiffness in lunate dorsal lobes is likely the expanded hemal spines we observed in the porbeagle, which create a flat plate of calcified material in the body of the tail. Ichthyosaurs are noted to have expanded neural spines arranged in a similar plate-like fashion which may also help to stiffen the anterior body and tail stock, but neural spines in the fluke are almost uniformly short and upright and will contribute little to tail stiffness (Buchholtz 2001).

It has been suggested that the fiber winding of collagen in the skin, which stiffens the body during swimming (Wainwright et al. 1978), also serves to stiffen tails (Motta 1977; Lingham-Soliar 2005). A broad comparison of dermal fiber angle between lunate and heterocercal tails has yet to be undertaken, though studies indicate that the overall fiber angle is greater in lunate tails than heterocercal tails, which may serve to additionally increase fin stiffness (Motta 1977; Lingham-Soliar 2005). Dermal collagen fibers have been noted in ichthyosaur fossils (Lingham-Soliar 1999, 2001; Lingham-Soliar and Wesley-Smith 2008), presumably acting as an exotendon (as in sharks) providing increased stiffness to the body and tail during swimming.

The blue shark exemplifies that not all pelagic tails are lunate or especially stiff (Riede 2004). While the blue shark is pelagic, its tail is neither lunate nor noticeably less flexible than other heterocercal tails. It has been suggested that sharks with lunate tails are not actually more energetically efficient swimmers than those with heterocercal tails, at least in juveniles (Blake 2004). Because the distinction between thunniform and carangiform swimming exists along a continuum, it may be that blue shark swimming kinematics are closer to thunniform than other species with heterocercal tails. Moreover, the lack of a stiffened tail may be overcome during active swimming. Both the blue shark and the porbeagle showed a greater degree of ceratotrichia overlapping neural spines than other species. Coupled with attachment to the radialis muscle, this may help to stiffen tails during active swimming (Flammang 2010). The apparent lack of adaptations for fast, sustained swimming in the blue shark may also be indicative of the functional trade-offs associated with such specializations. Tuna, and likely lamnids, have a large turning radius compared with other fish, making them less maneuverable (Blake 2004). Maneuverability will be affected by cross-sectional shape and body fineness ratio, and Carcharhiniforms, such as the blue shark, have morphologies that are conducive to greater maneuverability (Weihs 1981; Motani et al. 1996; Porter et al. 2009).

Thresher sharks are also pelagic, but have highly modified tails, used to hunt prey (Oliver et al. 2013). Unlike the porbeagle or the blue shark, the thresher ceratotrichia occur in three compartments, the first which runs over the neural spines and parallel to the long axis of the dorsal lobe; the second of which is angled caudally and overlaps the entire height of the neural spines; and the last of which is sparse, lies just over, and runs parallel to the centra. The second compartment is most similar to the ceratotrichia of other species. The first compartment of ceratotrichia likely acts similar to the cables of suspension bridge, to bear the tensile loads associated with the tail slapping behavior thresher sharks use when hunting (Oliver et al. 2013).

There are a number of studies examining the effects of fin flexibility and morphology on swimming ability (Lauder et al. 2011; Esposito et al. 2012; Leftwich et al. 2012; Shelton et al. 2014). However, these models may be misleading as they do not account for variability in stiffness within a single tail, either along the length of the dorsal lobe or between the dorsal and ventral lobes. Flammang et al. (2011) showed that the active control of tail position and stiffness affect flow around the tail in ways that cannot be predicted by a simple model foil. It may be that incorporating asymmetric lobe stiffness can account for some portion of this disconnect. This is especially important for studies that use modern sharks to explain swimming mechanics of extinct species, like ichthyosaurs (Massare 1988; Taylor 1987; Motani et al. 1996; Buchholtz 2001; Motani 2005; Lindgren et al. 2013; Molnar et al. 2015). Not only should these studies be mindful of the potential differences in stiffness between tail morphologies, but between lobes with skeletal support and those without (McGowan 1992).

Although ichthyosaurs can broadly be subdivided into groups with superficial similarities to carcharhiniform and lamniform sharks, the degree to which shark swimming mechanics can be used to inform hypotheses regarding ichthyosaur locomotion is limited. Sharks are negatively to neutrally buoyant, and there is a vertical component to the forces produced by their tail (Ferry and Lauder 1996; Wilga and Lauder 2002, 2004; Flammang et al. 2011). The classical theory held that this creates an overall downward torque for the head, counteracted by pectoral fins (McGowan 1992), or relative body angle (Wilga and Lauder 2002). More recent work has shown that, through kinematics and active control of position and stiffness, sharks do not produce lift with their pectoral fins (Wilga and Lauder 2000) can exert control over the pattern of wake vortices produced by the tail (Flammang et al. 2011). In contrast, ichthyosaurs were likely positively buoyant, like cetaceans, and are thought to have actively counteracted this while swimming (McGowan 1992; Taylor 2000; Lindgren et al. 2018). This similarity to cetaceans has led to the conclusion that ichthyosaur tails would be best modeled after cetacean or scombroid tails, with lobes of equal stiffness and no vertical component to the tail stroke (McGowan 1992).

We believe that it is fundamentally erroneous to assume that ichthyosaurs would not generate accessory forces to stabilize buoyancy; on the contrary, the dorsal-oriented heterocercal tail of the shark actively counteracting the negative buoyancy of the body is exactly the same problem in reverse as the ventral-oriented heterocercal tail of ichthyosaurs counteracting positively buoyant air-filled lungs and blubber. And perhaps more importantly, the dorsoventrally flexing cetacean body would have very different hydrodynamic effects when compared with the laterally undulating shark tail, especially with consideration of added mass and leading-edge vortex generation. Given these observations, sharks seem a likely candidate for functional morphological comparisons to ichthyosaurs.

This is not to say that comparisons should not be made to cetaceans. The skeletally-unsupported dorsal lobe of ichthyosaur tails may be more similar to cetacean flukes than shark ventral lobes, as ichthyosaurs do not have ceratotrichia. Cetacean flukes are composed of a core of crossing fibers surrounded by a ligamentous layer with bundles of fibers arrayed along the spanwise axis of the fluke, which serves to maintain spanwise rigidity while allowing some deflection to aid in swimming (Gough et al. 2018). In the absence of ceratotrichia and considering other soft-tissue similarities between ichthyosaurs and cetaceans (Lindgren et al. 2018), this may be a better analog for the composition of the dorsal lobe of ichthyosaur caudal fins.

While sharks may not serve as the only modern analog for understanding ichthyosaur locomotion, there is still much to be gleaned from understanding the structure and function of their asymmetrically supported caudal fins. Understanding the variation in stiffness (both active and passive) in both lobes, how they deflect under loads during swimming, and incorporating these findings into functional models will allow us to experimentally test hypotheses about ichthyosaur locomotion.

## Supplementary data

Supplementary data available at *IOB* online.

## Supplementary Material

Supplementary Data
