# Supplementary material for: Flexibility of Heterocercal Tails: What Can the Functional Morphology of Shark Tails Tell Us about Ichthyosaur Swimming?
Source: Integr Org Biol. 2019 Feb 19;1(1):obz002. doi: 10.1093/iob/obz002 (PMC7671117; doi:10.1093/iob/obz002)
Supplement: Supplementary Data [file obz002_supp.zip › Supplemental Table 1.docx]

Supplemental Table 1 – Basic morphology of all specimens studied

| Species | n | Tail Length (cm) | Tail Height (cm) | Tail Surface Area (cm^2^) | Aspect Ratio | Tail Angle (°) | Age | Sex |
| --- | --- | --- | --- | --- | --- | --- | --- | --- |
| *Alopias vulpinus* | 2 | 159.071 | 34.953 | 1788.873 | 0.683 | 164.166 | Juvenile | F |
| (common thresher shark) |  | 94.983 | 22.294 | 643.294 | 0.773 | 165.492 | Juvenile | M |
| *Carcharhinus brevipinna*  (spinner shark) | 1 | 33.069 | 25.388 | 255.425 | 2.523 | 145.831 | Juvenile | M |
| *Carcharhinus falciformis*  (silky shark) | 1 | 52.187 | 51.741 | 881.906 | 3.036 | 151.888 | Adult | F |
| *Carcharhinus obscurus* | 3 | 80.971 | 41.452 | 1337.102 | 2.681 | 167.927 | Adult | M |
| (dusky shark) |  | 57.138 | 45.654 | 777.481 | 2.681 | 160.117 | Juvenile | F |
|  |  | 21.11 | 18.164 | 126.28 | 2.617 | 164.397 | Juvenile | M |
|  |  |  | Species Average: | | 2.658 | 164.147 |  |  |
| *Carcharhinus plumbeus*  (sandbar shark) | 1 | 44.542 | 35.674 | 521.542 | 2.44 | 150.889 | Juvenile | F |
| *Carcharius taurus*  (sandtiger shark) | 1 | 29.239 | 13.181 | 206.364 | 0.842 | 174.227 | Juvenile | F |
| *Carcharodon carcharias* | 4 | 56.239 | 107.87 | 2597.457 | 4.48 | 139.091 | Adult | M |
| (white shark) |  | 54.142 | 93.962 | 2245.815 | 3.931 | 146.453 | Adult | M |
|  |  | 41.322 | 54.443 | 1058.075 | 2.801 | 143.881 | Juvenile | M |
|  |  | 32.112 | 37.576 | 486.87 | 2.9 | 153.936 | Juvenile | M |
|  |  |  | Species Average: | | 3.528 | 145.162 |  |  |
| *Isurus oxyrinchus* * | 2 | 45.069 | - | - | - | 139.752 | Adult | F |
| (shortfin mako shark) |  | 19.15 | 23.572 | 190.876 | 2.912 | 143.162 | Juvenile | M |
| *Lamna nasus* | 4 | 58.54 | 68.305 | 1229.348 | 3.795 | 152.13 | Adult | F |
| (porbeagle shark) |  | 51.079 | 72.726 | 1361.115 | 3.886 | 132.341 | Adult | F |
|  |  | 20.623 | 29.823 | 252.042 | 3.529 | 156.766 | Juvenile | M |
|  |  | 19.48 | 21.721 | 148.038 | 3.187 | 141.026 | Juvenile | M |
|  |  |  | Species Average: | | 3.599 | 145.566 |  |  |
| *Prionace glauca* | 4 | 66.557 | 45.402 | 1007.148 | 2.047 | 144.577 | Adult | M |
| (blue shark) |  | 38.226 | 19.751 | 289.957 | 1.345 | 159.125 | Juvenile | F |
|  |  | 23.245 | 13.588 | 103.237 | 1.788 | 163.711 | Juvenile | F |
|  |  | 21.028 | 9.658 | 78.801 | 1.184 | 165.865 | Juvenile | F |
|  |  |  | Species Average: | | 1.591 | 158.12 |  |  |

*Specimen M527 missing ventral lobe
