# Supplementary material for: Flexibility of Heterocercal Tails: What Can the Functional Morphology of Shark Tails Tell Us about Ichthyosaur Swimming?
Source: Integr Org Biol. 2019 Feb 19;1(1):obz002. doi: 10.1093/iob/obz002 (PMC7671117; doi:10.1093/iob/obz002)
Supplement: Supplementary Data [file obz002_supp.zip › Supplemental Table 2.docx]

Supplementary Table 2 – Summary of morphological data from dissected archetypes. Tails of each individual have been divided into subunits, each 10% of the length of the dorsal lobe from the centrum to the tip of the tail. For each subunit we have recorded: the number of centra, the ratio of average height of the rostral edge of the centra to average height of the caudal edge of the centra, the ratio of average length of the dorsal edge of the centra to average length of the ventral edge of the centra, ratio of centrum width to height for the rostral-most centrum of the portion as a representative, ratio of average centrum length to height, the average angle of neural spines relative to the dorsal edge of the centra, and the average angle of the hemal spines relative to the ventral edge of the centra.

| Species | % Length | # Centra | Rostral Height: Caudal Height | Dorsal Length: Ventral Length | Centrum Width: Centrum Height | Centrum Length: Centrum Height | Average Neural Spine Angle | Average Hemal Spine Angle |
| --- | --- | --- | --- | --- | --- | --- | --- | --- |
| *Alopias vulpinus* | 0-10 | 16 | 1.000 | 0.992 | 1.138 | 0.328 | 77.724 | 80.819 |
|  | 10-20 | 18 | 1.040 | 1.034 | 1.066 | 0.339 | 70.313 | 81.576 |
|  | 20-30 | 19 | 1.002 | 0.986 | 1.266 | 0.429 | 61.650 | 69.541 |
|  | 30-40 | 21 | 1.022 | 1.027 | 1.189 | 0.474 | 62.586 | 73.319 |
|  | 40-50 | 24 | 1.008 | 1.009 | 1.202 | 0.592 | 54.051 | 75.458 |
|  | 50-60 | 23 | 1.003 | 0.975 | 1.420 | 0.709 | 50.888 | 77.358 |
|  | 60-70 | 24 | 0.973 | 0.923 | 1.596 | 0.816 | 51.559 | 79.585 |
|  | 70-80 | 23 | 0.991 | 1.010 | 1.350 | 0.800 | 50.567 | 83.557 |
|  | 80-90 | 30 | 1.031 | 1.014 | 1.651 | 0.962 | 54.708 | 85.066 |
|  | 90-100 | 32 | 0.982 | 1.047 | 1.731 | 1.270 | 62.219 | 74.863 |
| *Carcharhinus obscurus* | 0-10 | 6 | 1.001 | 0.929 | 1.050 | 0.493 | - | 67.884 |
|  | 10-20 | 7 | 1.021 | 0.987 | 0.974 | 0.493 | 62.094 | 83.297 |
|  | 20-30 | 7 | 1.014 | 1.005 | 1.078 | 0.487 | 52.796 | 82.893 |
|  | 30-40 | 8 | 0.996 | 1.021 | 1.065 | 0.454 | 51.966 | 72.087 |
|  | 40-50 | 9 | 1.006 | 1.000 | 1.056 | 0.464 | 54.208 | 56.375 |
|  | 50-60 | 10 | 0.989 | 1.010 | 1.012 | 0.446 | 52.604 | 52.689 |
|  | 60-70 | 11 | 1.014 | 1.007 | 1.017 | 0.438 | 44.469 | 46.149 |
|  | 70-80 | 14 | 1.043 | 1.052 | 1.080 | 0.498 | 30.404 | 42.637 |
|  | 80-90 | 23 | 1.054 | 1.055 | 1.562 | 0.608 | 19.134 | 44.113 |
|  | 90-100 | 2 | 1.298 | 1.046 | 1.365 | 0.961 | 28.657 | 51.106 |
| *Carcharias taurus* | 0-10 | 6 | 0.989 | 1.049 | 0.267 | 0.617 | - | 61.051 |
|  | 10-20 | 6 | 1.048 | 1.048 | 0.426 | 0.665 | 48.533 | 74.529 |
|  | 20-30 | 7 | 1.033 | 1.033 | 0.574 | 0.660 | 48.376 | 74.101 |
|  | 30-40 | 7 | 1.018 | 0.996 | 0.717 | 0.676 | 44.345 | 66.739 |
|  | 40-50 | 8 | 0.980 | 0.987 | 0.943 | 0.689 | 46.318 | 56.627 |
|  | 50-60 | 8 | 0.989 | 1.002 | 1.161 | 0.707 | 52.621 | 46.118 |
|  | 60-70 | 10 | 0.976 | 0.993 | 1.585 | 0.729 | 48.142 | 45.642 |
|  | 70-80 | 12 | 1.014 | 0.977 | 2.183 | 0.755 | 42.515 | 45.017 |
|  | 80-90 | ~ 20 | 0.965 | 1.018 | 3.854 | 1.061 | 35.298 | 43.326 |
|  | 90-100 | 0 | - | - | - | - | - | - |
| *Lamna nasus* | 0-10 | 6 | 1.008 | 0.891 | 1.011 | 0.439 | - | 80.460 |
|  | 10-20 | 6 | 1.040 | 0.985 | 1.090 | 0.451 | 58.151 | 117.998 |
|  | 20-30 | 6 | 1.022 | 1.042 | 1.084 | 0.517 | 44.872 | 54.275 |
|  | 30-40 | 5 | 1.036 | 1.017 | 1.024 | 0.551 | 44.558 | 38.595 |
|  | 40-50 | 6 | 1.014 | 1.023 | 0.976 | 0.571 | 46.915 | 31.764 |
|  | 50-60 | 7 | 1.035 | 1.007 | 1.012 | 0.557 | 32.229 | 39.121 |
|  | 60-70 | 7 | 1.034 | 1.039 | 1.062 | 0.583 | 26.833 | 43.101 |
|  | 70-80 | 8 | 1.010 | 0.999 | 1.148 | 0.656 | 24.771 | 43.076 |
|  | 80-90 | 11 | 1.056 | 1.013 | 1.248 | 0.748 | 29.387 | 41.386 |
|  | 90-100 | 3 | 1.006 | 0.971 | 1.114 | 0.799 | 21.936 | 54.429 |
| *Prionace glauca* | 0-10 | 7.5 | 1.112 | 0.964 | 1.453 | 0.568 | 67.752 | 72.604 |
|  | 10-20 | 8 | 1.004 | 0.979 | 1.072 | 0.442 | 46.925 | 77.611 |
|  | 20-30 | 10 | 1.005 | 1.012 | 1.024 | 0.442 | 43.976 | 62.664 |
|  | 30-40 | 8 | 1.014 | 0.999 | 1.015 | 0.464 | 41.444 | 50.289 |
|  | 40-50 | 10 | 1.024 | 0.990 | 1.022 | 0.502 | 45.605 | 44.614 |
|  | 50-60 | 10 | 1.016 | 1.043 | 1.073 | 0.534 | 44.020 | 49.493 |
|  | 60-70 | 11 | 1.009 | 1.000 | 1.010 | 0.565 | 34.520 | 50.178 |
|  | 70-80 | 15 | 1.043 | 1.023 | 1.155 | 0.658 | 22.202 | 46.578 |
|  | 80-90 | 27 | 1.012 | 1.029 | 1.461 | 0.637 | 14.819 | 40.368 |
|  | 90-100 | 0 | - | - | - | - | - | - |
